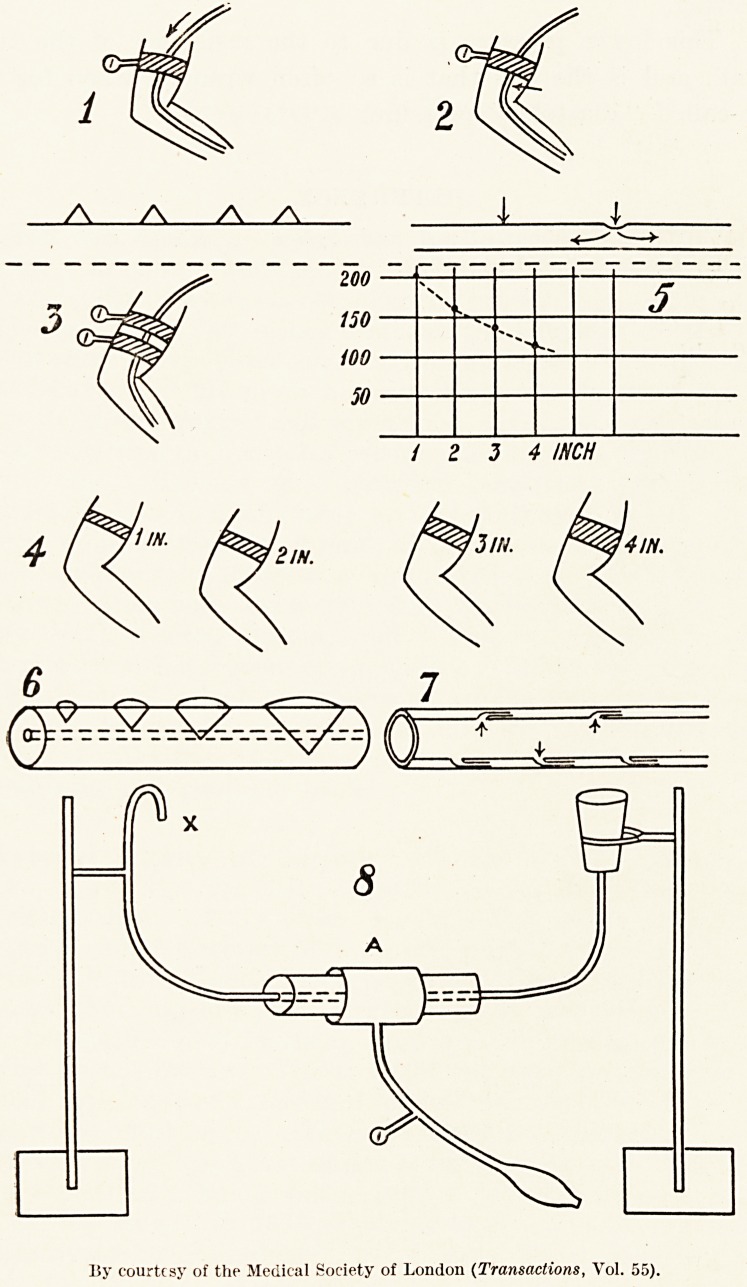# The Need for a Standard Method of Estimating the Blood-Pressure

**Published:** 1933

**Authors:** G. Arbour Stephens

**Affiliations:** Consulting Cardiologist King Edward VII Welsh National Memorial Association


### THE NEED FOR A STANDARD METHOD OF ESTIMATING THE BLOOD-PRESSURE.

BY

G. ARBOUR STEPHENS, M.D.,

*Consulting Cardiologist King Edward VII Welsh National Memorial Association.*

---

Two facts stand out very prominently in connection with the present method of estimating the blood-pressure.

The first is that the estimation of the systolic pressure varies with the width of the armlet, and the second is that the figure given as the *normal* diastolic or basic figure varies from 55 mm. to 85 mm. Hg., according to the observer.

As I have already pointed[1] out, the normal systolic pressure estimated by means of a 4-in. armlet equals 120 mm., with a 3-in. armlet it equals 135 mm. and a 2-in. armlet gives the figure as 150 mm. Hg.

So long as the width of the armlet be given it is immaterial what is the width, because the figures can all be reduced to a common denominator, but in my opinion it is far better to have one size of armlet common to all sphygmomanometers.

The marked variation in the figures given as the normal diastolic or basic pressure is of far greater consequence. For if the experts differ to such an extent, how can the ordinary practitioner hope to arrive at anything like a useful figure ?

The systolic pressure is a measure of the force exerted by the heart pump. But, in addition, we must know the resistance against which the work of that pump has to be done, if we are to arrive at any prognosis with regard to the heart. To estimate merely the systolic pressure is most misleading ; for although the systolic pressure be low, the resistance against which the heart has to work (which is measured by the basic pressure) may have been raised. Even when the basic pressure remains normal, and the systolic pressure falls, the ratio of one to the other is considerably modified, and this must be taken into account.

This point becomes more evident if we take actual figures, as in the following cases. By my method the normal figures are 150/50. If, then, the pressures are 100/50, it follows that the weakened heart has to pump against a resistance as great as the normal heart has to overcome. If the pressures are 150/80 the "normal" heart in this case has to work against an increased resistance (measured by a basic pressure of 80 instead of 50), and naturally tends to fail. This is the state of things which occurs in coronary thrombosis, and can only be estimated by taking both the systolic and the basic pressures.

It is necessary to realize that when the pressures are 150/80, in order to overcome the increased peripheral resistance the systolic pressure needs to be 240 mm. Hg. to maintain the circulation of the blood.

Under such an increased pressure the blood cells, although driven round the circulatory system, are seriously handicapped in their work, because the pressures are so high. Blood cells, both red and white, need an optimum pressure under which to live, move, and have their being satisfactorily, and to do their work properly. This pressure is one of 150/50 mm. Hg. Any increase or disturbance of that ratio affects their surface tension and consequently their osmotic processes, together with their interaction with the cells of the tissues.

My method of estimating the blood-pressures is as follows:—

1. Place the armlets (2 in. by 11 in.) of two sphygmomanometers on one arm, the one below the other.

2. Inflate the upper armlet until the pulse ceases to be felt, when the reading on the upper dial gives the systolic pressure.

3. Inflate forthwith the second armlet until the needle on the upper dial is moved upwards a point, when the reading on the lower dial gives us the basic pressure.

My method is mechanical and objective, and requires the minimum of skill and judgment, whereas the auscultatory method depends on the capacity of the doctor to detect and discriminate between various sounds which are not readily heard.

There are limitations to the auscultatory method, such as :—

1. In cases of irregular hearts, when there is a difference of intensity between successive sounds.

2. The arm may be too thickly covered with fat.

3. There may be no artery large enough to auscultate.

4. Many doctors have no ear for differences of sound, and are unable to detect the right sound at the right time, even if there be a right sound.

FIG. 1.—Shows that the inflation of armlet gives an estimation of the pressure in the direction of the arrow.

FIG. 2.—Shows that when the armlet is inflated for such an estimation, pressure on the artery at the point of the arrow sends the dial needle upwards. This effect is shown in the lower diagram in FIG. 2, when the pressure sufficient to indent the wall is conveyed both upwards and downwards along the full artery.

FIG. 3.—Shows the position of the two armlets, which cannot be more than two inches wide, else they cannot both be applied at the same time.

FIG. 4 and FIG. 5.—Draw attention to the several widths of armlets and the corresponding differences in the estimations.

FIG. 6.—Is to explain how the same pressure in the several widths produces wedge-shaped pressures of different sizes.

FIG. 7.—Is to show how an increased pressure in the artery compresses the mouths of the *vasa vasorum*, and by so doing interferes with the entrance of the blood into these vessels for the supply of the arterial walls.

FIG. 8.—Is a diagram of an experiment which showed that whilst it took a pressure of 80 mm. Hg. in the armlet A to stop the water flowing at X, the pressure in the armlet A

By courtesy of the Medical Society of London (*Transactions*, Vol. 55).

had to be reduced to 60 mm. Hg. before the water restarted flowing.

This lower pressure is due to the resiliency of the tube wall, and is the one that is so often wrongly taken for the so-called " diastolic " pressure.

### REFERENCE.

[1] Arbour Stephens, *Heart and Spleen in Health and Disease;* H. K. Lewis & Co. Ltd., 1932.

## Figures and Tables

**Figure f1:**